# Ten critical questions for scaling up floating developments

**DOI:** 10.1038/s41467-026-77492-2

**Published:** 2026-09-03

**Authors:** Elizabeth A. MacAfee, Tjeerd J. Bouma, Margo A. van den Brink, Joep van der Zanden, Christiaan Weiler, Floor Spaargaren, Rutger de Graaf-van Dinther, Olaf Waals

**Affiliations:** 1 Department of Spatial Planning and Environment, Faculty of Spatial Sciences, University of Groningen, Groningen, The Netherlands; 2 Department of Estuarine and Delta Systems, NIOZ Royal Netherlands Institute for Sea Research, Yerseke, The Netherlands; 3 Department of Physical Geography, Faculty of Geosciences, Utrecht University, Utrecht, The Netherlands; 4 Offshore Department, MARIN, Wageningen, The Netherlands; 5 Blue Revolution Foundation, Delft, The Netherlands

**Keywords:** Water resources, Research management

## Abstract

Floating developments may offer climate-adaptive solutions to space scarcity and water management challenges in coastal, inland, and offshore contexts. We present ten research questions to explore the potential for safe, inclusive, sustainable and economically viable upscaling towards large-scale floating developments.

Developing novel strategies to deal with accelerated sea-level rise (SLR), land subsidence, and extreme weather is essential, especially in densely populated deltas, coastal areas, and small island developing states (SIDS). Rather than applying merely incremental strategies, facing these challenges requires a fundamental shift in how society relates to water, and potentially a widespread shift towards building on water^1,2^. Floating developments are hence gaining attention around the world as a potential way to adapt to higher water levels and address space scarcity, for instance by transforming water from a periphery of cities and ports to an integral part of urban space^3^. Important lessons for future upscaling can be learned from existing vernacular floating structures, including in Low- and Middle-Income Countries, while successful small-scale floating projects in protected inland water systems may provide a valuable proof-of-concept for future upscaling^4–6^. Still, the challenges of moving from small-scale pilots and vernacular developments to widespread larger-scale implementation remain substantial.

Floating construction has multiple potential advantages in comparison to traditional land reclamation, buildings on stilts or piles, or building on land^7^. For example, floating structures can adapt to fluctuating water levels, they can be easily decommissioned, relocated, or repurposed, while hazardous activities can be migrated away from populated areas, and natural flow of water can be mostly maintained. A recent technical study^8^ of suitable locations for very large floating structures around coastal cities worldwide found enough potential space to house 2.6 billion people (138,000 km^2^)^8^, mostly in and around cities in Africa and Asia. This is twice as many people as are predicted to live in coastal areas at high risk from flooding and SLR in 2050, and 55 times as much space as has ever been created via land reclamation projects^8^. Part of this potential space is currently used by other functions such as recreation, shipping, fishing, and nature reserves. However, even if only 10% of this potential space could be used for floating housing developments, 260 million citizens could find housing here.

While ambitious and utopian visions of floating “climatopias” are plentiful^9^, they often lack attention to context-specific challenges including integration of large-scale floating developments into social, spatial, technological, and ecological landscapes. In this paper we therefore take a holistic perspective on pathways to upscaling floating developments, and in this sense, we depart from technical literature on VLFS, mega-floats, and so on^10^. We define large-scale floating developments as an individual large platform or multiple linked platforms comprising at least 20 hectares, and we define upscaling as both an increase in the size of individual platforms and/or the overall number of floating developments. Upscaling from small-scale developments and pilot projects to implementation of large-scale floating developments is currently hampered by knowledge gaps in multiple fields. We identify 10 critical questions which urgently need addressing to facilitate upscaling of large-scale floating developments, while avoiding unintended negative consequences, enabling informed decision-making, and supporting innovation.

## 10 critical research questions about large-scale floating developments

**Fig. 1:**
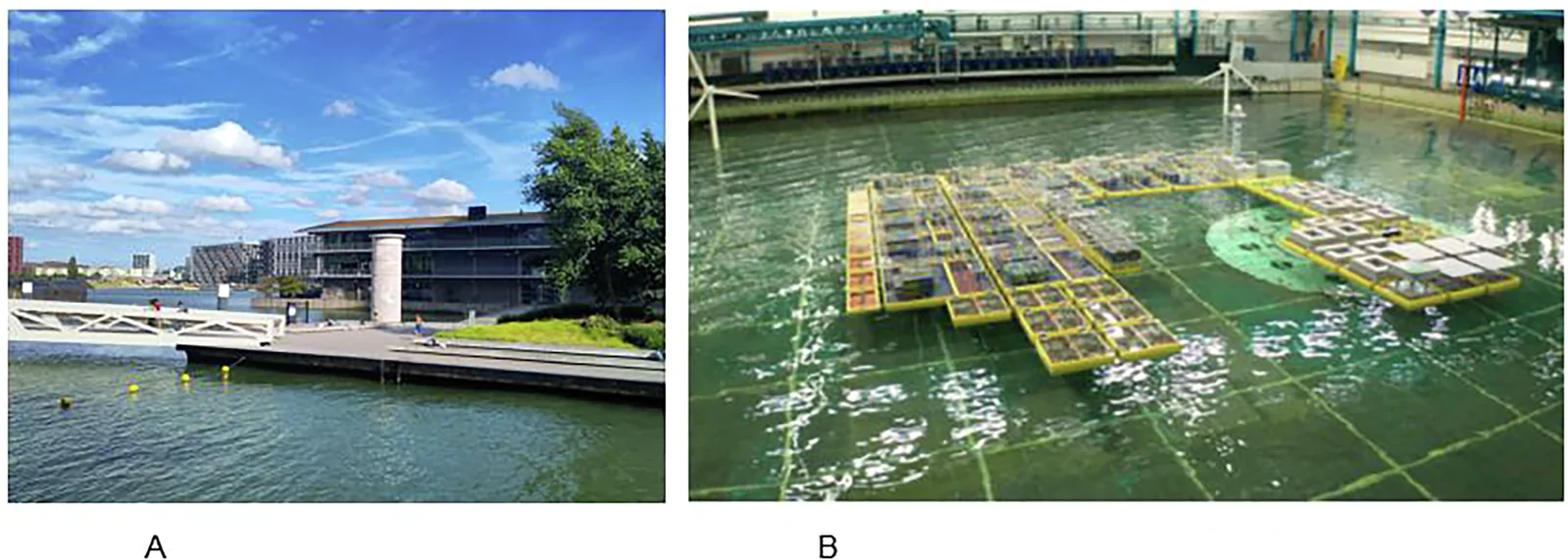
Images of floating developments. **A** Floating structures in Rotterdam, The Netherlands including the Global Center on Adaptation office in the background and a floating park in the foreground (Photo by Authors). **B** Model of an offshore floating industrial hub tested in the Space@Sea project (Photo used with permission by MARIN).

The 10 critical questions have been arranged according to the following topics regarding large-scale floating developments: (a) viability, including functions, locations, and business models (1-3); (b) feasibility in terms of technology, ecology, and governance (4-6); (c) desirability, including stakeholder perspectives, design guidelines and requirements, and environmental justice (7-9); and (d) pathways to upscaling (10) (Fig. 1). As a novel technology, the upscaling of floating developments needs to be studied from many angles, in relation to multiple scales, and with multiple stakeholders. The topics overlap and interact, both in how they should be studied scientifically and their implications in practice. Therefore, these questions can best be addressed in combination using interdisciplinary, creative, and participatory design and research methodologies to facilitate integration and collaboration with key stakeholders not only within, but also beyond, academia.

## Viability (1-3)

### What types of functions should large-scale floating developments provide?

Understanding the potential added value of large-scale floating developments requires analyzing what kinds of functions are most advantageous to have floating for societal, financial, or safety reasons. Current research has explored, for example, floating cities^11^ and floating ports^12^, where potential functions of floating developments may include but are not limited to: climate adapted and resilient housing^13,14^; energy production and storage; food production including aquaculture; maritime security; research activities; data centers; water desalination and purification; disaster relief; and nature and biodiversity conservation and restoration. The shift from small to large-scale floating platforms (or clusters of modular platforms) may facilitate some functions and be less suitable for others. Simultaneously, some activities are likely not technically or economically feasible to locate on a large floating platform, such as very tall buildings, railroads, or transfer of shipping containers. Further research is needed into defining suitability for the wide range of desired functions, including how to balance those functions and create synergies in mixed use or multifunctional floating facilities.

### How can suitable locations for large-scale floating developments be identified?

Multiple scientific studies have been conducted with the aim of identifying potential locations for floating developments, for example in relation to population pressure and sea level rise^15^ or environmental conditions like water depth, wave height, and wind. However, location selection should also take into account issues such as competition for space, distance from land, financial feasibility, social acceptance and readiness, and governance. Furthermore, the outcomes of these studies are typically fairly generic, while location selection criteria specific to different sized structures are also needed. Large-scale floating developments require significant amounts of space compared to existing small-scale developments, and much water already has a purpose (ie. recreation, shipping, fishing, and nature reserves).

### What business models can promote exploration of large-scale floating developments?

Developing large floating developments such as offshore floating multi-purpose platforms or floating neighborhoods requires new insights into affordability, societal added value, and the development of viable business cases^16^. In particular, more insight is needed into bottlenecks, opportunities, and risks from an investor perspective. Experimentation with innovative investment solutions, insurance, sustainable business models, and risk and financial assessment will also be required. For example, integrated life-cycle sustainability assessments consider multiple aspects of sustainability, including costs of maintenance and repair, environmental impacts, and longer-term societal benefit. Considering the long-term costs of maintenance and various externalities may make large-scale floating developments more attractive in the long term relative to land-based developments or reclamation projects.

## Feasibility (4-6)

### What additional technological innovations are needed for large-scale floating developments?

Technological innovations can support upscaling and expansion towards more exposed conditions while ensuring operability and survivability. Large floating developments encounter novel design challenges such as a complex hydro-elastic motion response, stress concentrations on connectors, and high global environmental loads. Key enablers are innovations that reduce global and mooring loads (e.g., dissipative floating breakwaters) or materials that increase durability. Civil engineers and urban designers will also face challenges in connecting civil infrastructure, such as sewage, power cables, and roads, to land or developing off-grid alternatives. For upscaling floating developments in industrial areas supply chain and logistics processes must also be considered.

### How can large-scale floating developments reach net-positive ecological impacts?

Any large-scale floating development will impact local ecology far more than current small-scale developments. Integrated knowledge of ecologists, civil engineers, and structural engineers will be needed to better understand the impacts of floating structures and how to mitigate them. For instance, the importance of scale in changes to light, shade, and hydrodynamics require further study, as do colonization by non-native species, corrosion, management of waste, and prevention of biofouling. Ideally, large-scale floating developments should reflect a commitment to promoting, protecting, and restoring life below water (Sustainable Development Goal 14). Studies are needed to examine whether and how large floating developments may enable recovery of lost biodiversity and ecosystem functioning, for example by hosting artificial reefs, integrating ecosystems along their (inner-)borders, or incorporating nature-based solutions.

### What kinds of governance arrangements do large-scale floating developments require?

Governance of large-scale floating developments is highly complex. First, it is not yet known how and to what extent large floating developments can be integrated into existing spatial policy, legislation, and regulations at the required national, regional, and municipal levels. Second, further research should explore the potential role of citizen participation (if developments are inhabited) in floating communities and societies. Governance arrangements will need to be context dependent to address specific legal, ownership, and insurance questions, including participation by governmental, civil-society, and market actors. These questions become even more complex in relation to the potential mobility and modularity of floating developments, or when floating development takes place in international waters or transboundary areas^17^.

## Desirability (7-9)

### What are potential stakeholders’ perspectives on large-scale floating developments?

Social and political acceptance of floating developments, including perspectives and values of current and potential future user groups, is not yet well understood^18^. Research on existing small-scale floating developments, including engineered floating villas, houseboat communities, and vernacular communities in Low- and Middle-Income Countries, can provide important insights into potential stakeholders’ perspectives on upscaling^6^, although the dynamics will likely be different as size increases. To extrapolate these studies to large-scale floating developments, participatory, futuring, and co-creation methods developed to study sustainability transitions and uptake of emerging technologies could be applied to study potential large-scale floating developments together with stakeholders who may be involved in or impacted by their implementation.

### How can existing design guidelines and requirements be adapted for large-scale floating developments?

Depending on the location and function of floating developments, design guidelines need to be established which incorporate multiple domains leading to integration of a variety of social, legal, technical, and ecological aspects. These can adapt and build on existing guidelines and requirements. For example, while the offshore industry already has thorough guidelines for environmental health and safety (EHS) in the maritime environment, large-scale floating developments and semi-permanent structures pose novel challenges related to floating-specific hazards^19^. For floating neighborhoods, existing criteria for new developments such as amounts and types of public space, safety and comfort, integration with existing cities, and infrastructure, utilities, and services, can be adapted to the floating or hybrid context. In addition, large floating developments may be located in places with multiple relevant governing bodies, meaning that a variety of design codes and EHS guidelines apply.

### What are the implications of large-scale floating developments for social and environmental justice?

Floating urbanization raises significant questions regarding social and environmental justice. Critical reflection is needed throughout research and development processes to avoid reinforcing social and spatial inequalities by catering to the needs of the wealthy, relying on exploitative supply chains, displacing or leaving behind marginalized groups, or causing enclosure of water which has previously been common public space. Social and spatial impacts of large-scale floating developments should be studied by scholars with knowledge about dynamics of densification, urban renewal, gentrification, and sustainable place-making. These challenges are already present with small floating developments and may potentially be exacerbated by upscaling - calling for novel strategies and solutions.

## Pathways to upscaling (10)

### What are possible pathways to upscaling floating developments?

Upscaling of floating developments could occur along multiple pathways. For example, upscaling might refer to continuity with business as usual, where large floating developments are mainstreamed into existing social, physical and institutional landscapes, spatial planning practices, and water management paradigms. Upscaling could also reflect a more transformative orientation to large floating developments as a novel way of living, working, and adapting to uncertain futures, reflecting a significant paradigm shift in how societies conceptualize their relationship with water^20^. How upscaling is framed by researchers and other stakeholders has important implications for how future large-scale floating developments may take shape, which is in itself an important topic for research. In addition, researchers studying large-scale floating developments should consider and be explicit about the normative assumptions and future visions underpinning their work.

Fifty years from now, within the typical lifespan of most coastal infrastructure, many major urbanized coastal areas and SIDS will face rising challenges from flooding and inundation^21^. Large-scale floating developments may provide complementary or alternative strategies alongside land reclamation and infrastructure for flood protection. If planned well and implemented carefully, large-scale floating developments can potentially avoid reproducing harmful conditions, functions, and attitudes from land^9^. Starting research proactively can enable realizing synergistic win-win opportunities, while avoiding negative externalities and impacts. Many steps are needed before ideas turn into feasible designs, pilot projects, bankable business models, and environmentally just and societally acceptable developments.

**Fig. 2:**
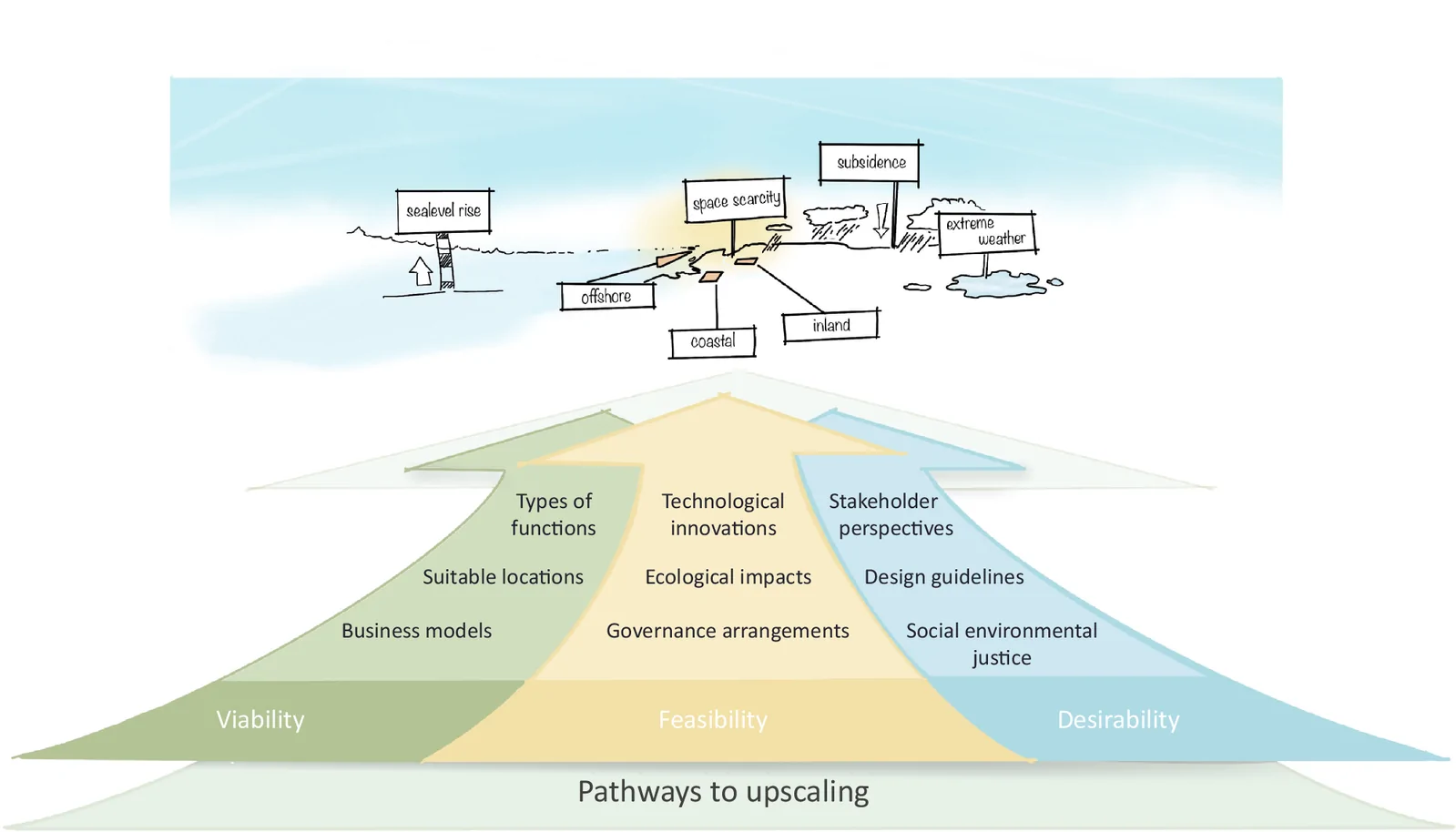
Integration of ten key-stone questions towards upscaling floating developments - The top portion of the figure shows possible locations of large-scale floating developments (coastal, inland, and offshore) and some drivers of upscaling floating developments (sea level rise, land subsidence, extreme weather, and space scarcity). The bottom portion of the figure has three arrows showing the categories of viability, feasibility, and desirability, with an overarching question about pathways to upscaling.
